# Phase II study on first-line treatment of *NIV*olumab in combination with folfoxiri/bevacizumab in patients with Advanced *CO*lo*R*ectal cancer RAS or BRAF mutated – NIVACOR trial (GOIRC-03-2018)

**DOI:** 10.1186/s12885-020-07268-4

**Published:** 2020-08-31

**Authors:** Angela Damato, Francesco Iachetta, Lorenzo Antonuzzo, Guglielmo Nasti, Francesca Bergamo, Roberto Bordonaro, Evaristo Maiello, Alberto Zaniboni, Giuseppe Tonini, Alessandra Romagnani, Annalisa Berselli, Nicola Normanno, Carmine Pinto

**Affiliations:** 1 Medical Oncology Unit, Azienda Unità Sanitaria Locale - IRCCS di Reggio Emilia, Oncologia Medica, Dipartimento Oncologico e Tecnologie Avanzate, Viale Risorgimento 80, 42123 Reggio Emilia, Italy; 2 Department of Medical Biotechnologies, University of Siena, Strada delle Scotte 4, 53100 Siena, Italy; 3 Azienda Ospedaliero - Universitaria Careggi, Dipartimento di Oncologia Medica, Largo G. Alessandro Brambilla 3, 50134 Firenze, Italy; 4 Istituto Nazionale Tumori IRCCS Fondazione G. Pascale, Dipartimento di Oncologia Addominale, Via Mariano Semmola 53, Napoli, Italy; 5 Istituto Oncologico Veneto I.R.C.C.S., S.C. Oncologia Medica 1, Dipartimento di Oncologia Clinica e Sperimentale, Via Gattamelata 64, 35128 Padova, Italy; 6 ARNAS Garibaldi – Azienda Ospedaliera di Rilievo Nazionale e di Alta Specializzazione Garibaldi, U.O.C. Oncologia Medica, Via Palermo 636, 95122 Catania, Italy; 7 Casa Sollievo della Sofferenza, Oncologia Medica, Dipartimento Onco-Ematologico, Viale Cappuccini 1, 71013 San Giovanni Rotondo, Italy; 8 Fondazione Poliambulanza Istituto Ospedaliero, U.O. Oncologia, Dipartimento Oncologico, Vial Leonida Bissolati 57, 25124 Brescia, Italy; 9 Policlinico Universitario Campus Bio-Medico, Oncologia Medica, Via Alvaro del Portillo 200, 00128 Roma, Italy; 10 Istituto Nazionale Tumori IRCCS Fondazione G. Pascale, Dipartimento della Ricerca, Via Mariano Semmola 53, Napoli, Italy

**Keywords:** Metastatic colorectal Cancer, First line therapy, Nivolumab, FOLFOXIRI Bevacizumab

## Abstract

**Background:**

FOLFOXIRI (fluorouracil, leucovorin, oxaliplatin, and irinotecan) plus bevacizumab has shown to be one of the therapeutic regimens in first line with the highest activity in patients (pts.) with metastatic colorectal cancer (mCRC) unselected for biomolecular alterations. Generally, tumors co-opt the programmed death-1/ligand 1 (PD-1/PD-L1) signaling pathway as one key mechanism to evade immune surveillance. As today, anti-PD-1 monoclonal antibodies are FDA approved only for DNA mismatch repair deficient/microsatellite instability-high (MMRd/MSI-H), which represent only about 5% among all mCRC. Nowadays, there are no data demonstrating anti PD-1 activity in proficient and stable disease (MMRp/MSS). A different target in mCRC is also the Vascular Endothelial Growth Factor A (VEGF-A), which acts on endothelial cells to stimulate angiogenesis. VEGF-A inhibition with bevacizumab has shown to increase the immune cell infiltration, providing a solid rationale for combining VEGF targeted agents with immune checkpoint inhibitors. Based on these evidences, we explore the combination of triplet chemotherapy (FOLFOXIRI) with bevacizumab and nivolumab in pts. with mCRC *RAS/BRAF* mutant regardless of microsatellite status.

**Methods/design:**

This is a prospective, open-label, multicentric phase II trial where pts. with mCRC *RAS/BRAF* mutated, in first line will receive nivolumab in combination with FOLFOXIRI/bevacizumab every 2 weeks for 8 cycles followed by maintenance with bevacizumab plus nivolumab every 2 weeks. Bevacizumab will be administered intravenously at dose of 5 mg/kg every 2 weeks and nivolumab intravenously as a flat dose of 240 mg every 2 weeks. The primary endpoint is the overall response rate (ORR). This study hypothesis is that the treatment is able to improve the ORR from 66 to 80%. Secondary endpoints include OS, safety, time to progression, duration of response. Collateral translational studies evaluate the i) tumor mutational burden, and ii) genetic alterations by circulating free DNA (cfDNA) obtained from plasma samples. The trial is open to enrollment, 9 of planned 70 pts. have been enrolled.

**Trial registration:**

NIVACOR is registered at ClinicalTrials.gov: NCT04072198, August 28, 2019.

## Background

The colorectal cancer (CRC) is diagnosed at advanced stages in almost 50%, and in this setting the 5-year survival rate is approximately of the 12% [1]. CRC is a heterogeneous tumor consisting of multiple genetic, genomic and epigenetic alterations, and this entails the stratification of the patients into different subgroups susceptible to different treatments. In 2015, a large-scale consortium reported four consensus molecular subtypes (CMS) of CRC described in MSI Immune (CMS1), Canonical (CMS2), Metabolic (CMS3), and Mesenchymal (CMS4) [2, 3], each with specific biomolecular and prognostic features.

In metastatic CRC (mCRC) the *RAS* gene (*KRAS, NRAS*) is mutated approximately in 50–55%. Currently, detection of *RAS* mutations is the only predictive marker of response to the anti-*EGFR* antibodies, cetuximab and panitumumab [4, 5].

The second potential biomarker in mCRC is *BRAF,* mutated in 5–11% of cases [6]. The *BRAFV600E* point mutation is the most common alteration and believed to be mutually exclusive with *KRAS* exon 2 mutations [7]. Accordingly, several clinical trials have highlighted the negative prognostic role of the *BRAF* mutation associated with high mortality [8].

In patients who harbor *RAS/BRAF* mutant tumors, the addition of anti-vascular growth factor (VEGF) antibody to cytotoxic drugs based on fluorouracil/levofolinate/irinotecan or oxaliplatin, has become one of the standard treatments in first-line of mCRC [9].

Several randomized studies, have proved that the triplet of chemotherapy with fluorouracil/levofolinate/irinotecan/oxaliplatin (FOLFOXIRI) combined to bevacizumab is more effective than doublet of chemotherapy plus bevacizumab, and this combination was well tolerated as first-line treatment in selected fit patients [10, 11]. In the TRIBE study [9], a phase III study, in first-line setting the treatment with FOLFOXIRI plus bevacizumab improved the primary endpoint, progression-free survival (PFS), compared with FOLFIRI (fluorouracil, leucovorin, and irinotecan) plus bevacizumab (HR 0.75; 95% CI 0.62–0.90; *p* = 0.003). A significant improvement and depth of tumor response associated with early tumor shrinkage assessed by Response Evaluation Criteria In Solid Tumors (RECIST) version 1.0, was also reported in experimental arm (FOLFOXIRI plus bevacizumab). Moreover, an advantage in terms of median overall survival (mOS) in FOLFOXIRI plus bevacizumab arm was revealed (29.8 months vs. 25.8 months; HR 0.80, 95% CI 0.65–0.98; *p* = 0.03). The molecular sub-analysis of the TRIBE study showed a better mOS in *RAS* and *BRAF* wild-type subgroups compared to *RAS* mutated and *BRAF* mutated subgroups (37.1 months vs. 25.6 months vs. 13.4 months), respectively [12].

In the VOLFI study [13], a phase II, patients affected by *RAS* wild type mCRC treated in first line with modified-FOLFOXIRI (m-FOLFOXIRI) plus an anti-EGFR antibody, panitumumab, presented a significantly improved the ORR (87.3%) compared to control arm (60.6%) both investigator and centrally assessment (95% CI, 1.61–12.38; *p* = .004). No difference in PFS was found (9.7 months in both arms, HR 1.071; 95%-CI 0.689–1.665, *p* = 0.76), but a strong trend about enhanced mOS in the experimental arm has been reported (35.7 months vs. 29.8 months; HR: 0.67; 95%-CI 0.41–1.11, *p* = 0.12) [14].

Currently, a further tumor feature being studied and of the great interest is the description of immune landscape of the microenvironment in mCRC, especially concern to microsatellite status. Most of tumors (85–90%) had a low-to-moderate mutation load and two main groups of CRCs were recognized: proficient in terms of mismatch repair mechanisms (MMRp) of DNA and microsatellite-stable (MSS). The minority are highly mutated with deficient mismatch-repair mechanisms (MMRd) relating to a microsatellite-instable phenotype (MSI, more accurately MSI-high [MSI-H]). This classification systems, MMRp/MSS vs. MMRd/MSI afford a way to stratify patients concerning to immunotherapy response [15, 16]. Nowadays, the immunotherapy approach with anti-PD1/PD-L1 antibodies for mCRC has demonstrated efficacy only in MMRd/MSI tumor subgroups but no in MMRp/MSS tumors. Several phase I-II-III clinical trials have been conducted (Table 1) and others are still ongoing (Table 2) to establish the immunotherapeutic efficacy alone or in combination with other drugs, especially with chemotherapy.

**Table 1:** Clinical Trials in mCRC of immune-checkpoint inhibitors as single agents or in combination

Drugs	Trial/Phase	Setting	Population	ORR n/N (%)	DCR n/N (%)	PFS (mo = months)	OS (mo = months)
**Pembrolizumab** [17]	NCT01876511 Phase II	> 2 L	MSI-H/dMMR	21/41 (52)	33/40 (82)	2-year = 59% mPFS NR	2-year = 85% mOS NR
**Pembrolizumab** [18, 19]	KEYNOTE-164 Phase II	> 2 L	MSI-H/dMMR	21/63 (33)	36/63 (57)	12-mo = 41% mPFS 4.1 mo (2.1 – NR)	12-mo = 76% mOS NR (19.2 – NR)
		> 3 L	MSI-H/dMMR	17/61 (28)	31/61 (51)	12-mo = 34% mPFS 2.3 mo (2.1–8.1)	12-mo = 72% mOS NR
**Pembrolizumab + mFOLFOX6** [20]	NCT02375672 Phase II	1 L	MSI-unselected	12/30 (40)	23/30 (77)	PFS not reported mPFS 16.9 mo (7.4, 16.9)	OS not reported mOS 8.8 mo (18.3-NE)
**Nivolumab** [21]	CheckMate 142 Phase II	> 2 L	MSI-H/dMMR	23/74 (31)	51/74 (69)	12-mo = 50% mPFS 14.3 mo (4.3, NE)	12-mo = 73% mOS, NR (18.0, NE)
**Nivolumab + low dose Ipilimumab** [22]	ChackMate 142 Phase II	> 2 L	MSI-H/dMMR	65/119 (55)	95/119 (80)	12-mo = 71% mPFS NR	12-mo = 85% mOS NR
**Atezolizumab** **+** **bevacizumab and fluoropyrimidine** [23]	NCT02291289 Phase II	1 L (maintenance)	MSI-unselected	Not reported	Not reported	mPFS 7.2 mo	mOS 22.1 mo
**Atezolizumab + FOLFOX + bevacizumab** [24]	NCT01633970 Phase Ib	> 2 L	Oxaliplatin naïve	9/25 (31)	Not reported	Not reported	Not reported
**Atezolizumab + bevacizumab** [25]	NCT01633970 Phase I	> 2 L	MSI-H/dMMR	4/10 (40)	9/10 (90)	mPFS NR (1.5–21.9)	mOS NR (2.6–23.7)

*ORR Overall response rate, *PFS* Progression free survival, *OS* Overall survival, *NE* Not estimable, *NR* Not reached, *m* Median

**Table 2:** Clinical Trials ongoing in mCRC of immune-checkpoint inhibitors as single agents or in combination with chemotherapy

Drugs	Trial	Setting	MSI/MSS status population	Primary Endpoint
**Nivolumab + standard therapy vs standard therapy** [26]	CheckMate 9X8 NCT03414983 Phase II/III	1 L	unselected	PFS
**Nivolumab alone** **Nivolumab in combination with other drugs** [27]	CheckMate 142 NCT02060188 Phase II	1 L	MSI/MSS	ORR by investigators
**Nivolumab + Ipilimumab + Temozolomide** [28]	NCT03832621 Phase II	1 L	MSS MGMT silenced	8-months PFS
**Nivolumab** **Nivolumab + Ipilimumab or standard therapy** [29]	NCT04008030 Phase III	1 L	MSI-H/MMRd	PFS
**Pembrolizumab vs standard therapy** [30]	KEYNOTE-177 NCT02563002 Phase III	1 L	MSI-H/MMRd	PFS, OS
**Pembrolizumab + pemetrexed and oxaliplatin** [31]	NCT03626992 Phase Ib	2 L+	MSS	ORR
**Atezolizumab vs atezolizumab + FOLFOX/bevacizumab vs FOLFOX/bevacizumab** [32]	COMMIT GI004/S1610 NCT02997228 Phase III	1 L	MMRd	PFS
**Avelumab** [33]	NCT03150706 Phase II	2 L+	MSI-H/MMRd POLE	ORR
**Avelumab vs standard chemotherapy +/− targeted therapy** [34]	NCT03186326 Phase II	2 L	MSI	PFS by central review
**Durvalumab** [35]	NCT02227667 Phase II	3 L+	MSI-I/MMRd	best response rate
**Durvalumab** [36]	NCT03435107 Phase II	2 L	MSI/MMRd POLE	ORR
**Durvalumab plus tremelimumab + FOLFOX** [37]	NCT03202758 Phase Ib/II	1 L	unselected	safety, PFS

*MGMT, O6-methylguanine-DNA methyltransferase; POLE, DNA polymerase epsilon, catalytic subunit

Preclinical studies, have shown the close connection between tumor cells and the tumor microenvironment (TME) status, especially the surrounding milieu composed by the stroma, tumor-infiltrating lymphocytes, and lymphatic and vascular layers. In this context, endothelial cells play a key role in the extravasation of immune cells, influencing the arrangement of the tumor environment [38]. It is known that extremely inflamed tumors reflect poor tumor angiogenesis; however, highly vascularized tumors may conversely entail tumors with deprived immune infiltration. One of the biomolecules responsible for affecting the hematopoietic progenitor cell differentiation to dendritic cells (DCs) is the tumor-derived VEGF. DCs are the most efficient antigen presenting cells due to the peptide presentation of tumor antigens on the major histocompatibility complex (MHC) I and II molecules, eliciting T-cells by B7 molecule expression, against cancer antigens [39, 40]. Active extravasation of leukocytes in the tumor stroma requires a series of events starting from the rolling, firm adhesion of leukocytes on endothelial cells, and leading to wandering into the interstitial areas. VEGF plays an essential role in this process as the blood vessels could present an obstacle to extravasation of immune cells in the interstitial space [41–43].

Additionally, VEGF inhibition by bevacizumab, involves a normalization of tumor vascularization rises the permeability to immune cell infiltration.

Given the strong preclinical rationale for combining VEGF inhibitors with immune checkpoint regulators, an increasing number of clinical trials are underway in several solid tumors including urothelial carcinoma [44, 45], metastatic renal cell carcinoma (mRCC) [46–48], and non-small cell lung cancer (NSCLC) [49–51], aiming to evaluate the anti-angiogenesis agents reinforce the benefit and durable responses afforded by anti- cytotoxic T-lymphocyte associated protein-4 (CTLA4) and the PD-1/PD-L1 agents.

It is essential to restore an immunological environment to sensitize mCRC to immune checkpoint inhibitors, to combine them with treatments that stimulate T-cells as chemotherapy, although the molecular mechanisms of sensitization are still not clear. Preclinical models suggest that some chemotherapies can improve the immunotherapy efficacy [52, 53]. The association of fluorouracil and oxaliplatin with immune checkpoint inhibitors in vivo could deplete Myeloid-derived Suppressor Cells (MDSCs) [54], and trigger an immunogenic arrangement of tumor cell death [55]. Dosset et al. [56], have investigated in two mouse models the use of FOLFOX in association with anti-PD-1 therapy. The combination induced a strong expression of PD-1 on CD8^+^ TILs, and the IFN-**γ** secreted by FOLFOX-induced CD8^+^ T cells leads PD-L1 expression on tumor cells and this mechanism is considered as an adaptive immune resistance system to FOLFOX. In neoadjuvant setting, mCRC patients treated with FOLFOX showed an increased CD8^+^ cell infiltrate and tumor PD-L1 expression. Another chemotherapeutic drug, trifluridine/tipiracil (FTD/TPI), an antimetabolite agent used to treat chemo-refractory mCRC, induced immunogenic arrangement of tumor cell death in vitro in MSS CT26 mouse colon carcinoma cell line, as well as in various human MSS colorectal cancer cell lines [57]. In vivo, the combination of FTD/TPI with oxaliplatin was able to induce immunogenic arrangement of tumor cell death, but not the single agents. Furthermore, the combination abolished type-2 tumor-associated macrophages (TAM2), resulting in higher cytotoxic CD8^+^ T-cell infiltration and activation. This effect was associated with tumor cells PD-L1 expression and PD-1 induction in CD8^+^ T cells, resulting in T-cell exhaustion.

Based on these preclinical and clinical data, there is sufficient evidence to explore the combination of chemotherapy with immunotherapy and antiangiogenetic inhibitors in pts. with mCRC *RAS/BRAF* mutated.

## Methods

### Protocol overview/study treatment

This is a prospective, open-label, multicentric phase II trial in which pts. with *RAS* or *BRAF* mutated will receive nivolumab in combination with FOLFOXIRI/bevacizumab as first line treatment. Study screening will take place within 28 days prior to initiation of study treatment. At screening, every patient must have local *RAS/BRAF* known status. A centralized review of *RAS/BRAF* status will be performed.

**Fig. 1:**
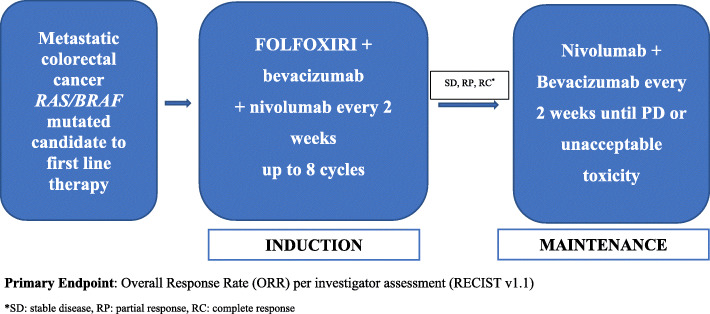
Study Design. Primary Endpoint: Overall Response Rate (ORR) per investigator assessment (RECIST v1.1). *SD: stable disease, RP: partial response, RC: complete response

Eligible pts. will be enrolled and begin treatment with FOLFOXIRI/bevacizumab plus nivolumab every 2 weeks for 8 cycles followed by maintenance with bevacizumab plus nivolumab every 2 weeks until disease progression, unacceptable toxicity or patient/physician decision. Bevacizumab will be administered intravenously at dose of 5 mg/kg every 2 weeks. Nivolumab will be administered intravenously at flat dose of 240 mg every 2 weeks. FOLFOXIRI will be administered as 165 mg/m^2^ intravenous infusion of irinotecan for 60 min, followed by an 85 mg/m^2^ intravenous infusion of oxaliplatin given concurrently with leucovorin at a dose of 200 mg/m^2^ for 120 min, followed by a 3200 mg/m^2^ continuous infusion of fluorouracil for 48 h (Fig. 1).

During the protocol’s treatment, pts. will be followed for safety based on Adverse Event (AE) assessments including vital signs, physical findings and clinical laboratory test results.

In order to guarantee the safety of pts., the enrolment will be stopped when the 10th patient will start treatment. An Independent Monitoring Committee will evaluate the safety data of these pts. and will decide if the study should be completed, amended or closed.

The efficacy will be evaluated by the investigator according to RECIST 1.1 criteria every 8 weeks during treatment, and then every 3 months for 3 years.

During the study baseline tumor blocks will be centrally analyzed to determinate MSI/MSS and PD-L1 status, inflammatory infiltrate through evaluation of high peri- and/or intra-tumor lymphocyte infiltration (TIL) using CD3^+^ and CD8^+^ TILs, tumor-associated macrophages (TAMs), tumor-associated neutrophils (TANs), and regulatory T cells (Tregs) well as the expression of marker of autophagy.

Following discontinuation of the treatment, safety assessments will be conducted 30 days after the last drug administration or until initiation of other anti-cancer therapy. Thereafter, pts. will be followed for disease progression (unless this has already occurred), serious AEs, anticancer therapy and survival. Follow-up will continue for up to 3 years.

A blood sample will be collected at baseline, prior to cycle 5, at the end of chemotherapy and at disease progression. Quality of life will be assessed at baseline, every 4 weeks during treatment and study discontinuation visit.

A list of participating centers is provided in Table 3.

**Table 3:** Participating Centers

Principal Investigator	Site	City
**Carmine Pinto**	Azienda USL – IRCCS Reggio Emilia	Reggio Emilia
**Francesca Bergamo**	Istituto Oncologico Veneto	Padova
**Evaristo Maiello**	Casa Sollievo della Sofferenza	San Giovanni Rotondo
**Alberto Zaniboni**	Fondazione Poliambulanza	Brescia
**Lorenzo Antonuzzo**	Azienda Ospedaliera Careggi	Firenze
**Guglielmo Nasti**	Istituto Nazionale Tumori di Napoli – IRCCS Pascale	Napoli
**Giuseppe Tonini**	Policlinico Universitario Campus Biomedico	Roma
**Roberto Bordonaro**	ARNAS Garibaldi – Azienda Ospedaliera di Rilievo Nazionale e di Alta Specializzazione Garibaldi	Catania

### Inclusion criteria

For inclusion in the study, all of the following inclusion criteria must be fulfilled: (i) histopathological confirmed colon adenocarcinoma; (ii) initially unresectable metastatic colorectal cancer not previously treated with chemotherapy for metastatic disease; (iii) assessment of *RAS* and *BRAF* status of the primary and/or secondary colon cancer on biopsies (mutant); (iv) age ≥ 18 years and ≤ 75 years; (v) ECOG performance status 0–1; (vi) if dihydropyridine dehydrogenase (DPD) status is known it must be wild type; (vii) laboratory data including: white blood cell count ≥3 × 10^9^/L with neutrophils ≥1.5 × 10^9^/L, platelet count ≥100 × 10^9^/L, hemoglobin ≥9 g/dL (5,6 mmol/l), total bilirubin ≤1.5 x ULN (upper limit of normal), ASAT and ALAT ≤2.5 x ULN, alkaline phosphatase ≤1.5 x ULN, serum creatinine ≤1.5 x ULN; (viii) signed written informed consent obtained prior to any study specific screening procedures.

### Exclusion criteria

Patients are not eligible for this study if any of the following exclusion criteria apply: (i) prior chemotherapy, excluded pts. treated in neo/adjuvant setting at least 12 months before diagnosis of metastatic disease; (ii) radiotherapy to any site within 4 weeks before the study; (iii) evidence of bleeding diathesis or coagulopathy; (iv) uncontrolled hypertension and prior history of hypertensive crisis or hypertensive encephalopathy (v) systemic corticosteroids within 2 weeks of the first dose of nivolumab; (vi) diagnosis of immunodeficiency or is receiving systemic steroid therapy within 14 days prior to the first dose of trial treatment; (vii) active and untreated brain (CNS) metastases and/or carcinomatous meningitis or subjects with previously treated brain metastases may participate provided they are not using steroids for at least 7 days prior to trial treatment; (viii) evidence of interstitial lung disease, active non-infectious pneumonitis, or a history of grade 3 or greater pneumonitis; (ix) live vaccine within 30 days prior to the first dose of trial treatment; (x) significant vascular disease (e.g. aortic aneurysm requiring surgical repair or recent arterial thrombosis) within 6 months of study enrollment; (xi) history of abdominal fistula, gastrointestinal (GI) perforation, intra-abdominal abscess or active GI bleeding within 6 months prior to the first study treatment; (xii) pregnancy (absence to be confirmed by ß-hCG test) or breast-feeding period; (xiii) any significant disease which, in the investigator’s opinion, would exclude the patient from the study.

### Study endpoints

The present trial will determine if adding nivolumab to the first line therapy with FOLFOXIRI/bevacizumab is efficient in terms of response rate in mCRC *RAS/BRAF* mutated. To evaluate the Overall Response Rate (ORR), defined as complete response (CR), partial response (PR), and stable disease (SD), we will use RECIST version 1.1 criteria.

Secondary endpoints are the following: (i) safety assessment of the combination treatment with FOLFOXIRI/bevacizumab plus nivolumab graded by National Cancer Institute (NCI) Common Terminology Criteria for Adverse Events (CTCAE) v. 4.03; (ii) OS defined as the time from beginning of the study-drug administration to the date of death from any cause; (iii) Time To Progression (TTP) defined as the time from beginning of the study-drug administration and the first date of documented progression, based on investigator assessment as per RECIST 1.1 criteria, or death due to any cause, whichever occurs first; (iv) the duration of response defined as the time between the first evidence of response (SD/PR/CR) and the date of documented progression or death due to any cause; (v) the quality of life of pts. determinate with the EORTC QLQ-C30 that consists of 30 questions that assess five aspects of patient functions (physical, emotional, role, cognitive, and social), three symptom scales (fatigue, nausea and vomiting, pain), global health and/or quality of life, and six single items (dyspnea, insomnia, appetite loss, constipation, diarrhea, financial difficulties) with a recall period of the previous week. Scale scores can be obtained for the multi-item scales.

The collateral study includes the TMB, MSI status and the role of genetic and molecular pattern analysis in relation to patient’s outcome. Formalin-fixed and paraffin-embedded (FFPE) tumor samples will be collected before starting fist-line therapy (at baseline), as primary and/or metastatic tumor tissue blocks or as 15 5-μm unstained slides. The neoplastic cell content of each tumor sample will be assessed and in those cases with neoplastic cells < 50% a macro-dissection of the specimen will be performed, if possible. For all the pts. enrolled, venous blood will be obtained by standard phlebotomy technique from a peripheral access point or from a central line, by trained personnel. Blood samples will be collected at different points: at baseline, prior to 5 cycle, at the end of chemotherapy and at disease progression.

### Data collection and follow up

Study drug administration occurs on Day 1 (± 3 days) of each cycle. Each cycle is 14 days. Cycle 1 should occur within 3 days from registration of pts. All procedures during the study treatment must occur within 3 days prior to the administration, except for radiological assessment required for baseline within 28 days prior to initiation of the study treatment. The following assessments will be performed prior to each cycle every 2 weeks. All radiological assessments will be performed each 8 weeks (± 1 week), regardless of the treatment cycle. CEA will be testing every 8 weeks with radiological assessments. The end of the study treatment visit should occur within 30 days after last dose of study treatment is administered. The post-treatment follow-up visits will occur every 3 months (± 14 days) for 3 years (Table 4).

**Table 4:** Study assessments

Procedures	Screening (−28 days)	Cycle 1,3,5,7^**a**^ (**+** 3 days)	Cycle 2,4,6,8 ^**a**^ (**+** 3 days)	Maintenance ^**a**^ (**+** 3 days)	End of treatment ^**a**^	Post-treatment Follow up ^**a**^
**Signed informed consent**	X					
**Medical history and baseline conditions**	X					
**Physical examination**	X	X	X	X	X	
**Parameters**^**b**^	X	X	X	X	X	
**Hematology and serum** **chemistry**^**c**^	X	X	X	X	X	
**Protein dipstick**	X	X	X	X	X	
**Adverse Events**	X	X	X	X	X	
**Radiological assessment**^**e**^**, CEA**^**c**^	X	X		X	X	
**QLQ-C30 questionnaire**^**f**^	X		X	X	X	
**Blood sample**^**g**^	X				X	
**FOLFOXIRI administration**		X	X			
**Nivolumab and Bevacizumab administration**		X	X	X		
**Survival follow-up**						X

^a^Each cycle is 14 days. Study drugs administration occurs on day1 (+/− 3 days) of each cycle. All clinical and laboratory assessments must occur within 3 days prior the administration. The end of treatment should occur within 30 days after last dose of study treatment. The post-treatment follow-up visit occur every 3 months (+/− 14 days) for 3 years

^b^Vital signs will include: weight, respiratory rate, pulse rate, temperature and systolic and diastolic blood pressure. At baseline height and BSA

^c^Hematology analysis (within 7 days before Cycle 1) consist of: hemoglobin, WBC and platelet count, BUN, creatinine, glucose, total bilirubin, sodium, potassium, calcium, AST, ALT, alkaline phosphatase, LDH, albumin. CEA will be tested every 8 weeks with radiological assessment. Amylase, lipase, TSH, FT3, FT4, will be done on cycle 2,4,6,8

^d^If proteinuria is 2+, should undergo a 24-h urine collection and must demonstrate 1 g of protein/24 h

^e^Radiological assessment will be performed within 28 days prior to start of study treatment and every 8 weeks (± 1 week), regardless cycle of treatment; in details, during chemotherapy phase prior to cycle 5, at the end of chemotherapy (cycle 8)

^f^QLQ-C30 will be completed at baseline, at cycles 4 and 8 of chemotherapy phase, every 4 cycles thereafter and at end of treatment visit

^g^Blood sample will be collected at baseline, prior to cycle 5, at the end of chemotherapy and at time of progression

### Statistical analysis and sample size

The primary objective of this study is to assess the ORR, defined as the best response recorded on the ITT population according to RECIST v1.1. In the TRIBE study, ORR for pts. *RAS/BRAF* mutated treated in first line with FOLFOXIRI and bevacizumab regimen was 66% [11]. Our hypothesis is that FOLFOXIRI and bevacizumab regimen plus nivolumab is able to improve the ORR from 66 to 80%. An ORR of 80% is considered enough valuable to pursue this combination in a phase III trial.

The sample size was calculated using the A’Hern [19] modification of the original Fleming [20] one-stage design. Calculations were performed by the use of PASS Professional v.11.0.10 software [21].

The study requires 64 subjects to decide whether the proportion responding, P, is less than or equal to 0,66 or greater than or equal to 0,80. If the number of responses is 49 or more, the Hypothesis that *P* < 0,66 is rejected with a target error rate of 0,05 and an actual error rate of 0,046. If the number of responses is 48 or less, the hypothesis that *P* > 0,800 is rejected with a target error rate of 0,200 and an actual error rate of 0,197. A total 70 pts. will to be enrolled assuming 10% pts. discontinuation rate due to non-compliance or toxicity.

### Preliminary safety evaluation

An Independent Monitoring Committee (IDMC) will review safety data 28 days after the inclusion of the 10th patient. Safety data, including demographics, adverse events, serious adverse events, and relevant laboratory data, will be reviewed.

The IDMC will provide a recommendation as to whether the study may continue, whether amendment(s) to the protocol should be implemented, or whether the study should be stopped. The final decision will rest with the Sponsor.

### Coordination

Azienda Unità Sanitaria Locale di Reggio Emilia – IRCCS is responsible for the coordination and management of the study on behalf of Gruppo Oncologico Italiano Ricerca Clinica (G.O.I.R.C.) Cooperative Group.

## Discussion

The binding of PD-L1 to PD-1 plays a central role in T-cell tolerance by hindering naive and effector T-cell responses. Clinical experience with checkpoint inhibitors has shown that tumors co-opt the PD-L1/PD-1 signaling pathway as one key mechanism to escape immune damage. Nivolumab, an anti-PD-1 monoclonal antibody may block tumor growth in different ways by targeting certain cells.

It’s well known that chemotherapy makes the cancer more immunogenic, and more suitable for immunotherapy. Moreover, angiogenetic inhibitors could promote enhanced tumor T-cell infiltration causing in a reprogramming of the tumor microenvironment from immune-suppressive to immune-permissive status. Novel anti-PD-L1 drugs reinforce the action of the antiangiogenetic drugs when administered in combination.

Encouraging early indicators of efficacy have been detected with combination strategies using immune-checkpoint inhibitors and biological targeted therapies, such as axitinib in combination with pembrolizumab [46], and nivolumab in combination with sunitinib or pazopanib [47] in mRCC. In another phase 1b study in mRCC, investigating the combination of bevacizumab and an anti -PD-L1, atezolizumab, increased intratumoral CD8^+^ T-cells and macrophages compared to bevacizumab alone, leading to an increase of MHC I expression, as well as Th-1 and T effector gene signatures in post treatment biopsies assessment [48]. Atezolizumab plus bevacizumab were examined in phase I, II and III studies. The safety of this combination resulted acceptable and AEs leading to treatment interruption were very low. In a phase III study, 40% of pts. treated with atezolizumab plus bevacizumab and 54% of pts. with sunitinib had grade 3–4 AEs; 12 and 8% of all-grade AEs led to discontinuation of treatment, respectively [48, 58, 59].

Recent findings for enhancement in PFS using bevacizumab and atezolizumab in combination with carboplatin/paclitaxel in front-line lung cancer is a promising strategy, indorsing clinically meaningful and durable benefit for patients [49–51].

In a clinical trial conducted in melanoma pts., has been explored the combination of bevacizumab with anti-CTLA-4 inhibitor, ipilimumab, revealed widespread morphological modifications in CD31^+^ endothelial cells and an extensive tumor penetration of immune cells post-treatment including CD8^+^ cells and CD163^+^ macrophages in comparison to ipilimumab treatment alone, thus demonstrating that the combination of anti-VEGF and anti-CTLA-4 inhibitors has the ability to promote immune cell access in the TME [60].

A recent phase III study revealed that in pts. with NSCLC, atezolizumab in addition to bevacizumab plus carboplatin and paclitaxel (ABCP) in 692 pts. with advanced non-squamous NSCLC improve OS (19.2 months vs. 14.7 months; HR 0.78; 95% CI, 0.64 to 0.96; *p* = 0.02) [51]. The safety profile of ABCP was consistent with safety profiles of each drugs and AEs occurred in 94.4% vs. 95.4% in ABCP and BCP control group, respectively. The most common grade 3 or 4 AEs were febrile neutropenia, and hypertension, and related serious AEs were noticed in 25.4 and 19.3% in the ABCP and BCP groups, respectively. The immune-related AEs (irAEs) grade 1 or 2 occurred in 77.4% of the ABCP group, and the treatment-related deaths occurred in 2.8% of the ABCP group [51].

In mCRC, a phase Ib study examined the safety and efficacy of atezolizumab plus bevacizumab (Arm A) with the dosage of atezolizumab 20 mg/kg q3w and bevacizumab 15 mg/kg q3w versus atezolizumab plus bevacizumab and mFOLFOX6 (Arm B) with atezolizumab 14 mg/kg q2w, bevacizumab 10 mg/kg q2w, and mFOLFOX6 at standard doses. The safety profile in Arm A showed a 64% of grade 3–4 AEs, while in Arm B, 73% pts. had grade 3–4 AEs, especially hematological toxicity. The irAEs grade 3 and 4 were 7 and 20%, respectively. The authors concluded that the addition of atezolizumab plus bevacizumab with or without FOLFOX was well tolerated without unexpected toxicities [61]. Efficacy data are not yet available.

In a phase II study, in 30 mCRC pts., pembrolizumab combined with mFOLFOX6 in first line treatment showed an acceptable toxicity thought suggesting a trend towards an increase of neutropenia; in the initial cohort grade 3 and 4 neutropenia was described but after dose reduction of mFOLFOX6, rate of grade 3 and 4 toxicity was 36.7 and 13.2% with FOLFOX/pembrolizumab and pembrolizumab alone respectively. Best response was partial response in 15 pts. with 100% of disease control rate (DCR) at 8 weeks. After 2 months of therapy, one patient with MMRd had surgical resection accounting complete pathological response. Moreover, the mPFS has not been reached [62].

In conclusion, we assume that there are sufficient evidences to support the combination of treatments with triplet chemotherapy (FOLFOXIRI), antibody anti-VEGF (bevacizumab), and immunotherapy (nivolumab, anti PD-1 antibody) in pts. with mCRC *RAS/BRAF* mutated, regardless to MMR status.

## Data Availability

Not applicable.

## Abbreviations

RAS: Rat sarcoma viral oncogene homolog
BRAF: V-Raf murine sarcoma viral oncogene homolog B1
FOLFOXIRI: 5-Fluorouracil, Oxaliplatin, Irinotecan
mCRC: Metastatic Colorectal Cancer
PD-1/PD-L1: Programmed death-1/ligand 1
MMRd/MSI-H: Mismatch repair deficient/microsatellite instability-high
MMRp/MSS: Mismatch repair proficient/microsatellite stable
VEGF: Vascular Endothelial Growth Factor
CMS: Consensus molecular subtypes
FOLFOX: 5-Fluorouracil, Oxaliplatin
CEA: Carcinoembryonic antigen
pts.: Patients
ECOG PS: Eastern Cooperative Oncology Group – performance status
NCI CTCAE: National Cancer Institute Common Terminology Criteria for Adverse Events
ORR: Overall Response Rate
CR: Complete Response
PR: Partial Response
SD: Stable Disease
OS: Overall Survival
PFS: Progression Free Survival
TTP: Time to Progression
RECIST: Response Evaluation Criteria in Solid Tumors
AE: Adverse Event
CI: Confidence Interval
HR: Hazard Ratio
FTD/TPI: Trifluridine/tipiracil
ICD: Immunogenic cell death
G.O.I.R.C.: Gruppo Oncologico Italiano Ricerca Clinica
